# Emergent Infectious Uveitis

**DOI:** 10.4103/0974-9233.58426

**Published:** 2009

**Authors:** Moncef Khairallah, Bechir Jelliti, Salah Jenzeri

**Affiliations:** Department of Ophthalmology, Fattouma Bourguiba University Hospital, Faculty of Medicine, Monastir, Tunisiati

**Keywords:** Infectious Disease, Systemic Illness, Uveitis

## Abstract

Infectious causes should always be considered in all patients with uveitis and it should be ruled out first. The differential diagnosis includes multiple well-known diseases including herpes, syphilis, toxoplasmosis, tuberculosis, bartonellosis, Lyme disease, and others. However, clinicians should be aware of emerging infectious agents as potential causes of systemic illness and also intraocular inflammation. Air travel, immigration, and globalization of business have overturned traditional pattern of geographic distribution of infectious diseases, and therefore one should work locally but think globally, though it is not possible always. This review recapitulates the systemic and ocular mainfestations of several emergent infectious diseases relevant to the ophthalmologist including Rickettsioses, West Nile virus infection, Rift valley fever, dengue fever, and chikungunya. Retinitis, chorioretinitis, retinal vasculitis, and optic nerve involvement have been associated with these emergent infectious diseases. The diagnosis of any of these infections is usually based on pattern of uveitis, systemic symptoms and signs, and specific epidemiological data and confirmed by detection of specific antibody in serum. A systematic ocular examination, showing fairly typical fundus findings, may help in establishing an early clinical diagnosis, which allows prompt, appropriate management.

## INTRODUCTION

Infectious causes should always be considered in all patients with uveitis. The differential diagnosis includes multiple well-known diseases such as herpes, syphilis, toxoplasmosis, tuberculosis, bartonellosis, Lyme disease, and others. However, clinician should be aware of emerging infectious agents as potential causes of systemic illness and also intraocular inflammation. Air travel, immigration, and globalization of business have overturned traditional pattern of geographic distribution of infectious diseases, and therefore one should work locally but think globally, though it is not possible always.

The purpose of this article is to review the systemic and ocular manifestations of several emergent infectious diseases relevant to the ophthalmologist including West Nile virus infection, rift valley fever, dengue fever, chikungunya and rickettsioses.

## WEST NILE VIRUS INFECTION

### Epidemiology

The West Nile virus (WNV), first isolated in 1937 in the West Nile district of Uganda, is an enveloped single-stranded RNA flavivirus. It is a member of the Japanese encephalitis virus serocomplex, which includes Japanese encephalitis virus, Saint Louis encephalitis virus, Murray Valley encephalitis virus, and Kunjin virus.^1^ The virus is widely distributed in Africa, Europe, Australia, and Asia, and, since 1999, it has spread rapidly throughout the Western hemisphere, including the United States, Canada, Mexico, and the Caribbean and into parts of Central and South America.^2^ West Nile virus infection is a zoonotic disease most often transmitted to human by an infected Culex mosquito vector with wild birds serving as its reservoir. The disease has been reported to occur anytime between July and December, with a peak onset in late summer. Other modes of transmission, including blood transfusion, organ transplantation, transplacental transmission, laboratory transmission, and breast feeding,^3^ have recently been recognized

### Systemic disease

The incubation period of WNV ranges from 3 to 14 days. About 80% of human infections are apparently asymptomatic. Only approximately 20% of persons infected become symptomatic with a self-limited febrile illness in most cases. Symptoms of affected patients include high-grade fever, headache, myalgia, arthralgia, malaise, nausea, vomiting, skin rash, weakness, and pharyngitis. The acute illness is self-limiting, typically lasting less than a week.

Severe neurologic disease (meningoencephalitis), frequently associated with advanced age and diabetes, was initially reported to occur in less than 1% of patients. However, over time, WNV infection has increased in severity.^1^–^3^

### Ocular disease

Since the first descriptions of ocular involvement secondary to WNV infection in 2002 and 2003, several ophthalmologic findings have been recognized, including chorioretinitis, anterior uveitis, retinal vasculitis, optic neuritis, and congenital chorioretinal scarring.^3^–^21^

#### Chorioretinitis

**Figure 1:**
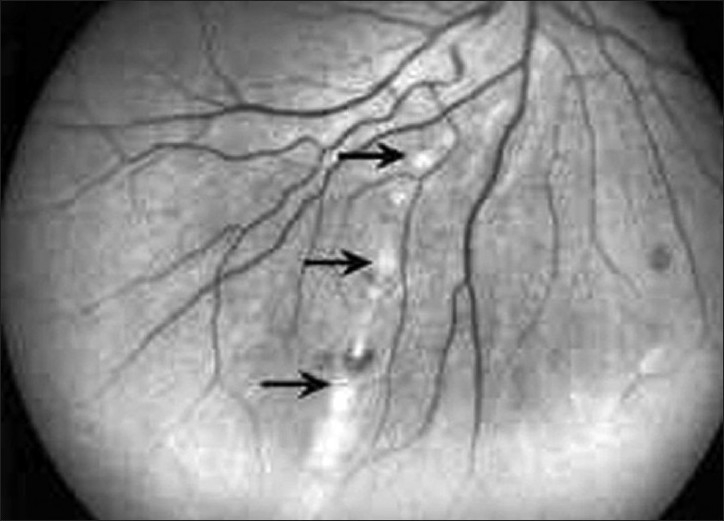
Red-free fundus photograph of the right eye of a patient with West Nile virus infection showing radial linear clustering of active deep creamy chorioretinal lesions (arrows)

**Figure 2:**
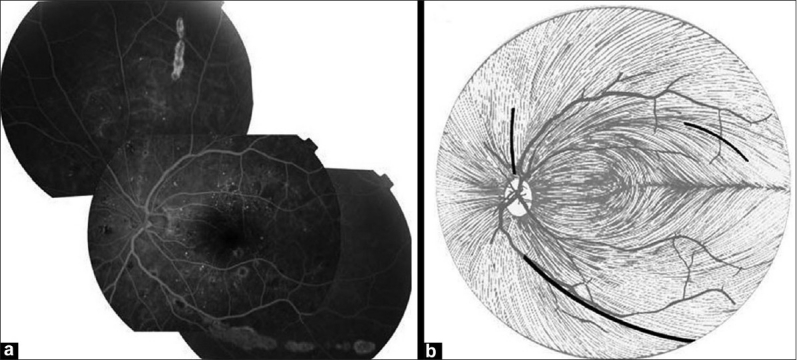
Midphase fluorescein angiogram (a) of the left eye of a diabetic 65-year-old woman with a history of WNV infection shows multiple linear streaks, which follow the course of retinal nerve fibres (b) Schema showing that linear streaks closely follow the course of retinal nerve fibres

A bilateral or rarely unilateral multifocal chorioretinitis, with typical clinical and fluorescein angiographic features, is the most common finding, occurring in almost 80% of patients with acute WNV infection associated with neurologic illness.^8^ Diabetes mellitus appears to be a potential risk factor for developing multifocal chorioretinitis, with more than 20% of patients having diabetic retinopathy in association with multifocal chorioretinitis. Most patients have no ocular symptoms or present with mildly reduced vision. An associated mild to moderate vitreous inflammation is frequently observed. The chorioretinal lesions commonly develop early in the course of the disease, appearing to be active (35%) or already inactive (65%) at presentation.^8^ Active chorioretinal lesions appear as circular, deep, creamy lesions on ophthalmoscopy [Figure 1], with early hyopofluorescence and late staining on fluorescein angiography. Inactive chorioretinal lesions are partially atrophic and partially pigmented with a “target-like appearance”: central hypofluorescence by blockage from pigment and peripheral hyperfluorescence [Figure 2a]. Some atrophic lesions are not pigmented. The lesions vary in number from less than 20 to more than 50 per eye. Chorioretinal lesions involve the midzone and/or pheriphery in almost all eyes. The posterior pole is involved in nearly 2/3 of eyes. Lesion size range from 100 to 1500 *μ*m, with most of the lesions measuring 200 to 500 *μ*m. Linear clustering of chorioretinal lesions is a prominent feature, occurring in more than 80% of eyes with chorioretinitis [Figure 2a]. The streaks vary in number, from one to more than three per eye, and in length approximately from 2 mm to 15 mm. They typically are oriented radially in the nasal and peripheral fundus or arranged in a curvilinear pattern in the temporal posterior fundus. The linear pattern of chorioretinitis appears to be related to the course of retinal nerve fibers, suggesting a contiguous spread of central nerve system disease [Figure 2b].^22^

Indocyanine green angiography shows well-delineated hypofluorescent choroidal lesions, which are more numerous than those appreciated by fluorescein angiography or clinically.^20^

Diabetes was found to be a risk factor for WNV-associated CR. It was also associated with more severe chorioretinal involvement.^23^ A recent prospective, controlled case series study emphasized the value of the typical multifocal chorioretinitis as a specific marker of WNV infection, particularly in patients who present with meningoencephalitis.^24^

#### Other ophthalmic manifestations

Although a typical multifocal chorioretinitis is the most common ocular manifestation of WNV infection, other findings can be observed. Anterior uveitis associated with vitritis in the absence of chorioretinitis has been reported.^11^ Numerous retinal vascular changes can occur, including retinal hemorrhages, focal or diffuse vascular sheathing, vascular leakage, and occlusive vasculitis.^3^,^8^,^13^,^14^ One patient with WNV infection had peripheral, segmental wedge-shaped zones of atrophy and mottling of the retinal pigment epithelium, which might be due to occlusion of ciliary artery.^8^ West Nile virus-associated optic nerve involvement may occur, including optic neuritis, optic disc swelling, and optic disc staining on fluorescein angiography.^8^,^15^–^18^ Other reported neuro-ophthalmic manifestations include ocular nerve palsy and nystagmus.^8^ A case of congenital chorioretinal scarring secondary to intra-uterine transmission of WNV infection has been reported.^19^

### Diagnosis

Diagnosis of WNV infection requires a high index of suspicion and specific laboratory testing. The most common efficient diagnostic method is detection of WNV-specific IgM antibody in serum or cerebrospinal fluid using the antibody-capture enzyme-linked immunoabsorbent assay. As IgM antibody does not cross the blood-brain barrier, its presence in the cerebrospinal fluid strongly suggests infection of the central nervous system. The plaque-reduction neutralization test can help in distinguishing false-positive results of MAC-ELISA or other assays as well as serologic cross-reactions among the flaviviruses.^1^

The unique pattern of multifocal chorioretinitis can help to establish an early diagnosis of the disease while serologic testing is pending. 

### Differential Diagnosis

The differential diagnosis of WNV systemic disease include other arthropod-borne viral encephalitides, enteroviral aseptic meningitis, herpesvirus encephalitis, encephalopathy from systemic illnesses (Legionnaires Disease, rickettsiose, Epstein-Barr virus infectious mononucleosis, and systemic lupus erythematosus), epidural abcess, hypertensive encephalopathy, and drug-induced meningitis.

Many infectious and inflammatory conditions may present with chorioretinitis. The differential diagnosis includes syphilis, tuberculosis, histoplasmosis, sarcoidosis, and idiopathic multifocal chorioretinitis.^3^ West Nile virus-associated chorioretinitis can be distinguished from these entities on the basis of history, systemic signs and symptoms, and particularly the unique pattern of chorioretinitis.

### Treatment

There is, at present, no proven treatment for WNV infection. In cases of severe systemic disease, intensive supportive therapy is indicated, often involving hospitalization, intravenous fluids, respiratory support, prevention of secondary infections, and good nursing care.

Antiviral agents such as ribavirin and interferon were found to be active only *in vitro.*^25^,^26^

Several clinical studies, including an open-label trial of interferon α-2b and a placebo-controlled trial using high-titer intravenous immunoglobulin, are ongoing.^27^

Prevention is the mainstay of WNV infection control: measures to reduce the number of mosquitoes (draining standing water, larvicides), personal protection (repellents, window screen, protective clothing). Vaccination, a long-term solution, is still in the research phase.^3^,^27^

Specific ophthalmic treatment may be required: topical steroids for anterior uveitis, peripheral retinal photocoagulation for neovascularization due to occlusive vasculitis, pars plana vitrectomy for non-clearing vitreous hemorrhage or tractional retinal detachment, and intervention with laser, photodynamic therapy, or intravitreal injection of anti-VEGF for choroidal neovascularization.^13^

### Evolution and prognosis

The outcome WNV systemic disease is good in most patients. However, severe cases may result in neurologic sequela or death, especially in patients who are elderly or debilitated.^1^,^2^

Ocular involvement usually has a self-limited course. Active chorioretinal lesions at presentation evolved to the typical inactive stage. Some inactive lesions become more prominent on both ophthalmoscopy and fluorescein angiography. Visual acuity returns to baseline in most patients. However, persistent visual impairment may occur due to a foveal chorioretinal scar, choroidal neovascularization, and complications of occlusive retinal vasculitis, such as vitreous hemorrhage secondary to retinal neovascularization, severe ischemic maculopathy, optic atrophy, or retrogeniculate damage.^3^,^12^,^13^,^15^,^16^,^20^

## RIFT VALLEY FEVER

### Epidemiology

Rift Valley fever (RVF) is an arthropod-borne viral disease caused by *Bunyaviridae*, primarily affecting domesticated cattle. It is transmitted to humans through a bite by infected mosquitoes or through direct contact with infected animals.^28^ The disease was first described in the Rift Valley of Kenya in 1930.^29^ Since, several outbreaks have been reported in sub-Saharan and North Africa, and more recently in Arabian Peninsula.

### Systemic disease

Human exposure to the virus is often occupational, either through handling infected livestock or their products or by breathing in aerosols released at slaughter. Mosquito bites and the consumption of raw milk have been documented as routes of exposure. The incubation period in humans is generally from 3 to 7 days, followed by one of three clinical syndromes.

The most common clinical syndrome is an uncomplicated, febrile, influenza like illness. The main symptoms are fever with biphasic temperature curve, headache, arthralgias, myalgias, and gastrointestinal disturbances.^30^ The fever subsides in 12 to 36 h, and the other symptoms are relieved within four days. Other clinical presentations include a hemorrhagic fever with liver involvement, thrombocytopenia, icterus and bleeding tendencies; and a neurologic involvement with encephalitis following a febrile episode with confusion and coma. Death is infrequent but there may be some residual damage.

### Ocular disease

**Figure 3:**
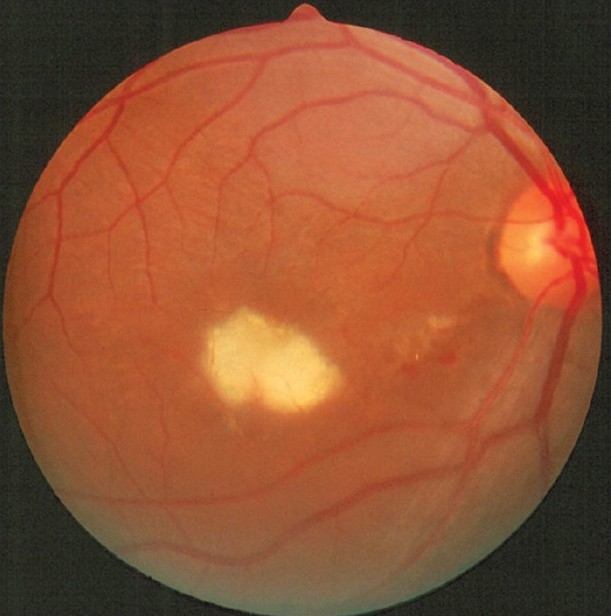
Fundus photograph of the right eye of a patient with Rift valley fever shows active retinitis in the macular region. The presence of retinal hemorrhages should be noted. (Courtesy: E Abboud)

Ocular involvement has been reported to occur in 1 to 20% of RVF infections.^29^,^31^–^36^ Al-Hazmi *et al*,^31^ recently reported the largest series (30 hospitalized patients and 113 outpatients) of serologically proven RVF with ocular manifestations during an outbreak in Saudi Arabia. The mean interval between the onset of RVF and visual symptoms ranged from 4 to 15 days. Macular or paramacular retinitis was identified in all the affected eyes at the time of initial assessment [Figure 3]. Other lesions included retinal hemorrhages (40%), vitreous reactions (26%), optic disc edema (15%), and retinal vasculitis (7%). Anterior uveitis was present in 31% of outpatients. Fluorescein angiography of the retinitis showed early hypofluorescence with late staining of retinal lesions and blood vessels. Symptoms resolved spontaneously within two to three weeks from the onset of systemic symptoms and did not result in complications such as glaucoma, posterior synechia, or cataract. Initial visual acuity was less than 20/200 in 80% of eyes in the outpatient group. Vision remained the same or deteriorated in 87% of eyes. Evaluation at the last follow-up showed macular (60%) or paramacular (9%) scarring, vascular occlusion (23%), and optic atrophy (20%) in the outpatient group.

### Diagnosis

Once an outbreak is recognized and early cases are proven, it becomes much easier to diagnose further cases of RVF. The most popular method of laboratory diagnosis uses serology to detect antibodies, either IgM antibodies in a single serum sample, or by detecting a rising titre of IgG antibody in acute and convalescent serum samples.^37^ In patients with encephalitis, cerebrospinal fluid may be tested for locally produced IgM. With the presence of equipped virological laboratories, RVF virus can be isolated from blood in the acute phase of illness. Finally, PCR detects the viral nucleic acid sequence of the RVF virus both in serum and tissue.^38^

### Differential diagnosis

The differential diagnosis for RVF retinitis includes other viral entities such as measles, rubella, influenza, dengue fever, and West Nile virus infection. These diseases can be differentiated from RVF by clinical history and serologic testing. Other hemorrhagic fever viruses have been reported to have ocular involvement such as Hantaan virus, Puumala, Marburg, and Ebola viruses.^39^

Some bacterial infections have been reported to cause retinitis. These mainly include rickettsial infection, Lyme disease, and cat scratch disease. These diseases can be ruled out with serologic testing.

### Treatment

Treatment is entirely supportive for mild to moderate cases of RVF simple analgesia and fluids can be administered. For patients who develop severe disease, including encephalitis, and hemorrhage, early recognition and aggressive critical care, including assisted ventilation and blood product transfusion is essential, for any hope of survival.^40^

Intensified mosquito control methods must be implemented in areas of epizootic and human RVF activity. An increased awareness of RVF among both residents and visitors to endemic areas is paramount to the future control and prevention of outbreaks. Education regarding modes of disease transmission and necessary precautions, especially protection against mosquito bites is vital.^41^

### Evolution and prognosis

Most cases of human RVF manifest as a mild self-limited influenza like illness.^40^ However, ocular involvement is frequently associated with permanent visual loss resulting from macular and paramacular scarring, vascular occlusion, and optic atrophy.

## DENGUE FEVER

### Epidemiology and systemic disease

Dengue fever is the most common mosquito-borne viral disease in humans. It is caused by the dengue virus, a flavivirus, of which there are four serotypes, and is transmitted by the Aedes aegypti mosquito. It is considered to be one of the most important arthropod borne disease in the tropical and subtropical regions, being endemic in more than 100 countries. It afflicts 100 million people annually thereby putting 2.5 billion people at risk worldwide.^42^

Dengue fever is endemic in Singapore but the number of cases has risen dramatically in the last few years.^43^ In addition to fever, dengue fever also causes headaches, myalgia and thrombocytopenia, resulting in bleeding manifestations such as a purpuric rash. Hypotension may also occur in the Dengue shock syndrome which carries a high mortality rate.^42^

More recently, dengue fever has also been found to affect the eyes, with resultant loss of vision.^44^–^49^

### Ocular disease

Ocular involvement in dengue fever has recently been reported from Singapore, Thailand, Taiwan, India, Mexico and Brazil. 44–54 In a cross-sectional observational study conducted in Singapore in 2005 during a dengue epidemic,^55^ we found that the prevalence of dengue maculopathy among patients hospitalized for dengue fever was 10%.

These patients tend to be young, with an average age of 29 years, ranging from 11 to 61 years.^50^ There is no sex or racial predilection.^50^,^55^

#### Subconjunctival hemorrhage

The most common ocular involvement reported in an East Indian population was a petechial type of subconjunctival hemorrhage (37%), which was associated with a platelet count of less than 50 000 *μ*l.^51^

#### Dengue maculopathy

This occurs as a result of involvement of the retinal and/or choroidal vessels, with a predilection for the macula area. Anterior segment involvement is usually mild and may be easily missed. The ocular symptoms may range from mild blurring of vision to catastrophic and severe blindness and they usually occur within a month of the onset of the dengue infection.

**Figure 4:**
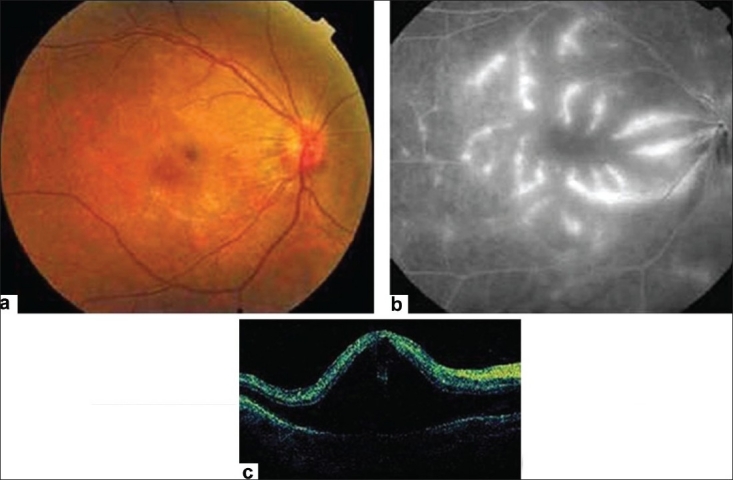
(a) Fundus photograph; (b) fluorescein angiogram; (c) and optical coherence tomogram of the right eye showing dengue maculopathy manifesting as severe retinal vasculitis with serous retinal detachment. Fundal examination showed macular hemorrhages, macular vasculitis with sheathing of predominantly the venules. The fundal fluorescein angiogram demonstrates prominent vascular leakage. optical coherence tomogram shows the presence of severe neurosensory retinal detachment. (Courtesy, Soon-Phaik Chee)

The fundal manifestations typically occur one week after the onset of fever, just as the fever has settled and the platelet count is recovering.^47^,^49^ The patients may present with a sudden decrease in vision (87%), a central scotoma (63%) or floaters (1%).^50^ The involvement is bilateral in 73% but tends to be asymmetrical.^50^ The mean initial best-corrected visual acuity was 20/45.^50^ The main fundal findings 50 were retinal hemorrhage (45%), venular sheathing (45%) yellow subretinal dots (28%), retinal pigment epithelium mottling (17%), round foveal swelling [foveolitis] (16%), disc hyperemia (14%), disc edema (11%), and arteriolar sheathing (4%) [Figure 4a]. There may also be cells in the anterior chamber (17%) and vitreous (11%).

Dengue maculopathy involves both the retinal and the choroidal circulation, hence fluorescein and indocyanine green angiography are important modalities in the assessment of its severity, as some of the changes may not be evident clinically and may therefore be missed.^50^

### Fluorescein angiography

The main FA findings^50^ include blocked fluorescence (33%), venular occlusion (25%), venular leakage (13%) [Figure 4b], knobby capillary hyperfluorescence (13%), retinal pigment epithelium (RPE) hyperfluorescence (10%) [Figure 4b], venular leakage with occlusion (5%), arteriolar leakage (3%), RPE window defect (3%), arteriolar occlusion (2%), capillary nonperfusion (2%)

Interestingly, in 3% of eyes, fundal fluorescein angiography (FFA) is unremarkable despite clinically observed venous sheathing on fundus examination.^50^ When the maculopathy involves larger retinal vessels, regardless of whether they are inflammatory or occlusive in nature, the visual acuity and field loss tends to be greater.^50^ Furthermore, eyes with occlusive vascular involvement tend to have residual scotomas, corresponding to depressed multifocal ERG recordings.^56^

### Indocyanine green angiography

Yellow subretinal dots appear as early RPE hyperfluorescence on FFA, but these changes are seen in only 15% of the cases. In comparison, ICG showed hypofluorescent spots during the mid to late phase, corresponding to these yellow dots as well as additional spots in areas without clinically evident dots in 29% of cases. The angiogram findings suggest that the yellow subretinal dots involve the choriocapillaries and/or RPE. These lesions resolve clinically without residual visual loss.^50^

Large choroidal vasculopathy with hyperfluoresence and leakage is also seen on ICG (31%) and is associated with mild reversible visual disability.^50^

Optical coherence tomogram (OCT) is indispensable in eyes with decreased visual acuity, but no apparent lesions can be observed on angiography. OCT is also useful for assessing the severity and monitoring the progress of neurosensory retinal detachment in 15% of eyes with retinal vascular leakage [Figure 4c]. Macular cystic spaces were starkly absent in macula edema associated with dengue maculopathy.^50^ OCT is able to image a corresponding area of focal outer neurosensory retina-RPE thickening at the foveal center. It is also useful for monitoring clinical progress.^50^

**Figure 5:**
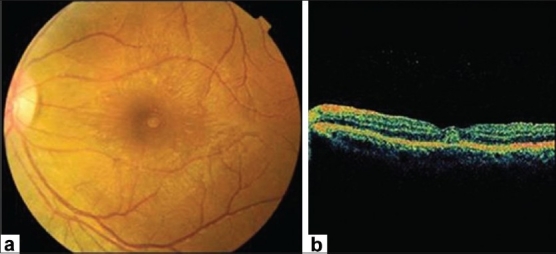
(a) Fundus photograph; (b) and optical coherence tomogram showing dengue foveolitis in the left eye. (c) before fundus. Fundus photograph showed an elevated orange spot at the fovea. The optical coherence tomogram demonstrated a focal thickening of the outer neurosensory retina-retinal pigment epithelial layer (Courtesy, Soon-Phaik Chee)

Foveolitis, clinically seen as a well-circumscribed pale yellowish lesion at the center of the fovea [Figure 5a], is best imaged with the OCT [Figure 5b], as FFA only rarely may demonstrate early foveal hyperfluoresence.

### Pathophysiology

The pathophysiologic mechanism of dengue maculopathy is still unclear. The average onset of ocular symptoms from the onset of illness is about seven days.^47^,^49^,^50^,^55^ This delay as well as the lower the serum complement C3 levels in patients with maculopathy as compared to those without maculopathy supports an immune-mediated mechanism rather than direct viral infection of the eye.^50^,^55^

### Diagnosis

The diagnosis of dengue fever is based on the typical clinical presentation of and a positive dengue IgM serology. The diagnosis of dengue maculopathy is based on clinical features as well as the imaging studies described below.

### Treatment

It is unclear whether treatment is beneficial or what is the optimal modality of treatment, as the disease may be self-limiting and there have been no prospective randomized trials on therapy to date. Immunosuppression with topical, periocular, oral, intravenous steroids and immune globulins has been attempted with variable success.^50^

## CHIKUNGUNYA

Chikungunya virus is an arthropod borne alphavirus in the family Togaviridae.^56^ It is responsible for the recent explosive epidemic in Indian Ocean region.^57^,^58^ This virus has three distinct genotypes, the East African, West African and Asian, maintained in monkeys and wildlife populations. Epidemics are sustained by human-mosquito-human transmission by several mosquito species including Aedes aegypti and A. albopictus. The incubation period is two-five days, disease may manifest about 48 h after mosquito bite.^56^ Chikungunya is usually a self-limiting febrile illness lasting for few days to weeks; most of the patients recover without consequences. However, the current outbreak seems to be more severe with increased morbidity and even significant mortalities.^57^–^59^ This review highlights all systemic and ocular clinical manifestations of the disease which were not seen in previous outbreaks.

### Epidemiology

Chikungunya virus was first isolated from the blood of a febrile patient in Tanzania in 1953. It is endemic in parts of west, central and southern Africa and few areas of Asia. Several outbreaks have been reported between 1957 and 1974, an explosive emergence of the disease occurred in island of Réunion in 2005.^60^ This outbreak is the most severe and one of the biggest outbreaks which has affected millions of the people in several islands in the Indian Ocean.^60^–^62^ The disease then spread to Seychelles, Madagascar, Comoro Islands, Mauritius, and Mayotte subsequently to Sri Lanka, Maldives, India, Malaysia and Indonesia. 57,59,60,63–65 Previous Indian outbreaks were caused by the Asian genotypes, while the 2006 epidemic is attributed to the East African genotype.^64^,^65^ In 2007 North-Eastern Italy reported new cases.^66^ Travelers returning from outbreak areas to Europe, Canada, the Caribbean and South America, United States, and Australia were found to suffer from chikungunya fever.^67^–^69^ Schuffenecker reported unique viral mutation, an emergence of a new genotype with an A226V in the membrane fusion glycoprotein E1which may be responsible for the enhanced adaptation in the vector.^70^ Explosive nature of chikungunya in the recent outbreak may also be associated this viral mutation, absence of herd immunity, lack of vector control, and globalization of travel.^69^–^72^

### Systemic disease

Chikungunya fever may manifest as an acute fever with headache, fatigue, myalgia, diffuse maculo papular rash, bleeding from the nose or gums, peripheral edema, and joint pain.^60^–^63^,^68^,^69^ The main clinical feature is a dramatic reduction in movement even to carry out routine daily activities due to impaired strength and pain in hands and legs. Prolonged fatigue, pronounced lethargy and depression last for several weeks.^68^ Asymptomatic infections do occur but the incidence is not known.^67^ The recent outbreak added neurological signs, vision threatening ocular complications, acute hepatic failure, multi organ failure and mother-to-child transmission.^57^,^59^–^63^,^71^

The name, chikungunya, origins from the posture of the patients in Makonde language of southeastern Tanzania, which means to become contorted. They characteristically lie still in the attitude of flexion because of severe joint pains.^63^ Joint pains are typically polyarticular and migratory, most severe in the morning.^73^–^75^ Three different types of rheumatic manifestations may coexist in same patient including finger and toe polyarthritis, severe tenosynovitis of wrists, hands, and ankles;^68^ and exacerbation of mechanic pain in previously injured joints.^73^–^75^ In some patients, an invalidating poly-arthralgia may persist for months or years.^76^ Radiological findings and biological markers of inflammation may remain normal.^73^

When pregnant women in Réunion had acute chikungunya infection within 48 h before delivery, neonates presented with an abrupt onset of fever, flushing of the skin, maculopapular rash, conjunctival congestion, pharyngitis, upper respiratory tract disease and meningoencephalitis within five days of birth. 61,71 Facial edema, gingivorrhagia, bullous rash, pronounced sloughing, and localized petechia seem to be frequent in children. Chikungunya virus may be found by PCR in blister fluid.^71^ The symptoms of disease in older children are same as adult patients with acute fever, headache, myalgia, and polyarthralgia.^76^

Neurologic manifestations of chikungunya virus infection was very rare previously,^77^ sero positive meningoencephalitis hs been reported both in adults and children in the present epidemic, some patients also had viral genome in the cerebrospinal fluid.^78^,^79^ As seen in several arboviruses, Guillain-Barre syndrome has been seen in serologically proven chikungunya virus patients.^77^ Other neurological signs include bilateral external ophthalmoplegia, upper motor neuron facial palsy, hemiparesis, incongruous homonymous hemianopias suggestive of optic tract lesions.^80^

### Ocular disease

To date, chikungunya was known to cause minor ocular signs such as photophobia, conjunctivitis and retro orbital pain. 56 However, more recent reports reveal both mild and vision threatening ocular complications.^80^–^84^ Ocular symptoms usually occur after a latent period of a month to year; however, few concurrent presentations have also been reported.^80^,^83^ It is either unilateral or bilateral, affecting both the genders in all age groups. Two distinct presentations are seen in this infectious uveitis including anterior and posterior uveitis.^80^–^84^

#### Anterior uveitis

Patients present with symptomatic uveitis including redness, pain, photophobia, blurred vision, and floaters. A mild granulomatous or nongranulomatous anterior uveitis, pigmented diffuse keratitic precipitates on the central or over the entire corneal endothelium and stromal edema may be seen on slit lamp examination.^81^–^83^ Posterior synechia is not common. These eyes develop uveitis-induced raised Intra ocular pressure with open angles even before steroid use. Anterior uveitis closely mimic herpitic viral keratouveitis, however, bilateral involvement is uncommon in herpetic uveitis. Significant iris pigment release in the anterior segment, past history of fever with and joint pain along with positive serologic results can differentiate this from herpitic disease. Visual prognosis in chikungunya anterior uveitis is good.^81^–^83^

#### Posterior uveitis

**Figure 6:**
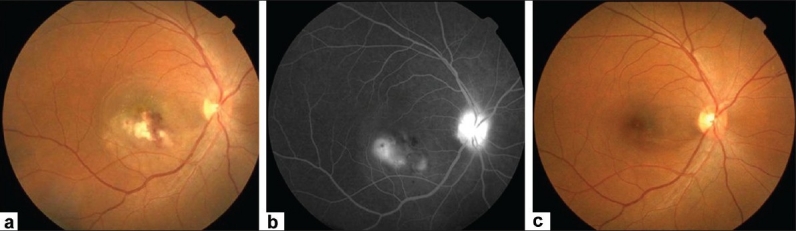
Fundus photograph (a) of the right eye of a patient with chikungunya showing neuroretinitis, multifocal retinitis and serous retinal detachment. Fluorescein angiography; (b) shows hyperfluorescence of the retinal lesion and optic disc leakage. Fundus photograph of the right eye taken six months after presentation; (c) shows resolution of fundus findings. The presence of temporal disc pallor should be noted. (Courtesy, Padmamalini Mahendradas)

Patients may present with history of loss of vision, color vision defect, central or centrocecal scotoma and peripheral field defects.^80^–^84^ Patients with posterior uveitis may have normal anterior segment and intraocular pressure is normal. Posterior pole or macular retinochoroiditis appear relatively more specific and carries very poor visual prognosis [Figure 6a,b and c]^81^,^82^,^84^ Chikungunya retinitis is similar to herpetic retinitis; however, markedly less vitreous reaction and confluent posterior pole retinitis can differentiate chikungunya from the acute retinal necrosis which is characterized by intense vitritis and peripheral multifocal or disseminated retinitis.^81^–^82^ Other posterior segment signs include optic neuritis, neuroretinitis and retrobulbar neuritis.^80^,^81^

To summarize, the individuals, with anterior uveitis with pigmented keratic precipitates or confluent posterior pole retinochoroiditis or optic neuritis or neuroretinitis and a history of fever with arthralgia who live in or come from endemic area for chikungunya virus, may have this entity included in the differential diagnosis.

### Differential diagnosis

Chikungunya is clinically very similar to dengue fever, West Nile fever, urban yellow fever and o'nyong-nyong fever. Although dengue and chikungunya are transmitted by the same vector, the incubation periods are dramatically different. In the ideal laboratory condition, the average incubation period for dengue ranges from 8 to 14 days, while for chikungunya it is 2 to 5 days. 65 Abrupt onset and shorter duration of fever with arthralgia are frequent in chikungunya, hemorrhagic manifestations are uncommon and as a rule shock is not observed.^63^,^85^,^86^ Decreased platelet count, severe hemorrhage and shock are characteristic of dengue, rashes occur in both the diseases but are more common in dengue. However, confirmatory diagnosis of chikungunya fever from dengue or O'nyong-nyong can be made only by the laboratory investigation.

### Laboratory tests

Complete hemogram reveals leucopoenia, lymphocytosis, mild thrombocytopenia, an elevated erythrocyte sedimentation rate and a positive C-reactive protein in acute cases.^56^,^65^,^87^ Three specific laboratory tests are virus isolation, serological tests and molecular techniques.^88^–^94^ Virus isolation and RT-PCR are useful during the initial viremic phase (day 0 to day 7) while serological methods are useful after 10 days of infection. However all three diagnostic modalities are not available in most of the places. The major challenge is to develop and standardize the diagnostic kits for regional hospitals to perform the diagnostic tests.^65^

### Virus isolation

Chikungunya virus produces cytopathic effects in a variety of cell lines including BHK-21, HeLa and Vero cells. The cytopathic effects must be confirmed by chikungunya specific antiserum and the results will be available in one to two weeks.^88^–^90^

### Serological diagnosis

Serologic diagnosis can be made by demonstration of fourfold increase in antibody in acute and convalescent sera or demonstrating IgM antibodies specific for chikungunya virus.^56^,^88^,^89^ A commonly used test is the IgM Antibody capture enzyme-linked immunosorbent assay.

### Molecular techniques

Reverse transcriptase polymerase chain reaction (RT-PCR) technique is used for specific detection of the virus amplifying fragment of E-2 gene.^92^ Combined detection and genotyping of chikungunya has been developed targeting nsP1 and E1 genes.^93^ More recently, specific and sensitive one-step TaqMan RT-PCR assay is available as a tool for diagnosis and rapid indicator of active infection by quantifying viral load in clinical samples and cell culture supernatant.^94^

### Treatment

#### Systemic treatment

Specific antiviral drug is not available; treatment is mainly symptomatic including rest, non steroidal anti inflammatory drugs and paracetamol. Patients with dehydration, who cannot take oral fluids, may need intravenous rehydration. Dramatic improvement is observed with corticosteroids, especially in the presence of refractory arthritis, tenosynovitis, nerve entrapment syndromes, or Raynaud phenomenon.^75^

#### Ocular treatment

As there is no specific anti viral drug, only topical steroid eye drops are given in case of simple anterior uveitis. Timolol maleate 0.5% eye drops twice daily and oral acetazolamide, 250 mg twice daily for three days are added with topical steroids when the patients come with uveitic glaucoma. Even though there is no evidence in the literature to support the efficacy of acyclovir or other antiviral agents against chikungunya, some investigators treat confluent retinitis with intravenous / oral acyclovir and oral prednisolone.^80^–^84^

### Prognosis

Chikungunya is considered to confer life-long immunity.^67^ In general, the disease is benign and self-limiting. Visual prognosis in chikungunya anterior uveitis is better than in the posterior form.

## RICKETTSIOSES

### Epidemiology

Rickettsioses are zoonoses due to obligate intracellular small Gram-negative bacteria. Most of them are transmitted to humans by the bite of contaminated arthropods, such as ticks. Rickettsial agents are classified into three major categories: the spotted fever group, the typhus group, and the scrub typhus.^95^,^96^

The spotted fever group includes Mediterranean spotted fever (MSF), Rocky Mountain spotted fever (RMSF), and numerous other rickettsioses. MSF, also called “boutonneuse” fever or tick-borne rickettsiosis, which is caused by the organism *Rickettsia (R.) conorii*, is prevalent in Mediterranean countries and Central Asia, including India. Rocky Mountain spotted fever, which is caused by *R. rickettsii*, is endemic in parts of North, Central, and South America, especially in the south-eastern and south-central United States. The other multiple rickettsial species belonging to the spotted fever group vary in their geographic distribution. Epidemic typhus, which is caused by the organism *R. prowazekii*, is usually encountered in areas of crowded population with poor hygiene conditions, as occurs during wars and natural disasters. Murine typhus, which is caused by *R. typhi*, is found worldwide in warm-climate countries. Scrub typhus, which is caused by *Orienta tsutsugamushi*, is a zoonosis found in the Far East.^95^,^96^

### Systemic disease

A rickettsial disease should be suspected, during spring or summer, in the presence of the triad of high fever, headache and general malaise, and skin rash in a patient living in or traveling back from a region endemic for rickettsioses. A local skin lesion, termed as “tache noire” (black spot) may develop at the site of arthropod bite in MSF and several other rickettsioses. A history of outdoor activities, occupational exposure, or tick attachment is frequent.^95^,^96^

### Ocular disease

Ocular involvement is common in patients with rickettsiosis, but as it is frequently asymptomatic and self-limited, it may be easily overlooked.^97^,^98^ However, rickettsial ocular disease may be associated with ocular complaints, such as decreased vision, scotoma, floaters, or redness. Retinitis, retinal vascular involvement, and optic disc changes are the most common ocular findings, but numerous other manifestations may occur.

#### Retinitis

**Figure 7:**
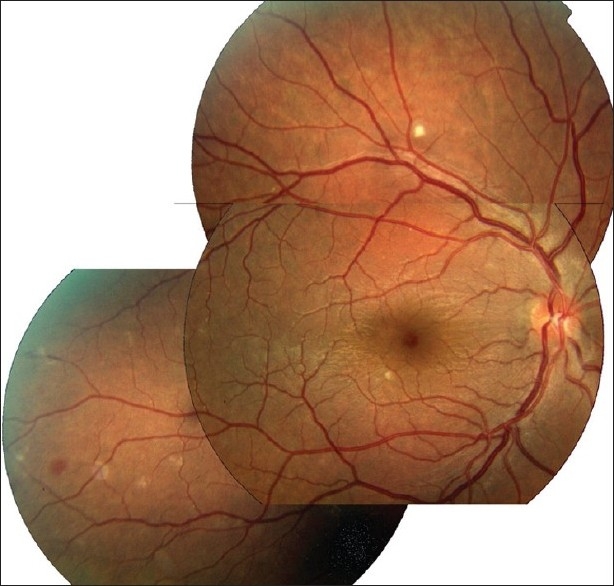
Fundus photograph of the right eye of a patient with Mediterranean spotted fever shows multifocal retinitis, with white retinal lesions of small size adjacent to retinal vessels

**Figure 8:**
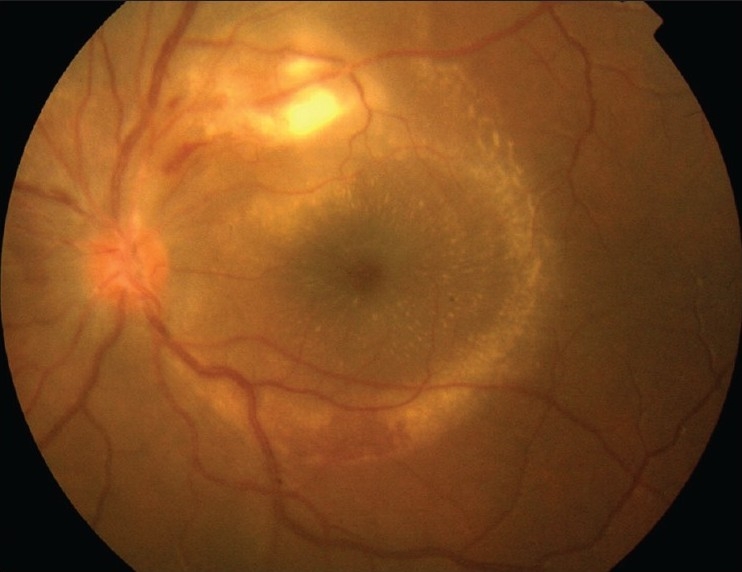
Color fundus photograph of the left eye of a patient with Mediterranean spotted fever shows a temporal juxtavascular white retinal lesion with associated serous retinal detachment and a macular star

It is observed in at least 30% of patients with acute MSF. 98 It presents in the form of white retinal lesions, typically adjacent to retinal vessels [Figure 7]. These lesions may vary in number, size, and topography. An associated mild or moderate vitreous inflammation is commonly observed. Fluorescein angiography showed early hypofluorescence and late staining of large acute white retinal lesions and isofluorescence or moderate hypofluorescence of small active retinal lesions throughout the whole phase of dye transit.^98^ OCT shows serous retinal detachment, accurately detected by optical coherence tomography (OCT), frequently accompanies large foci of rickettsial retinitis [Figure 8].

There are reports of multiple small white retinal lesions in other rickettsioses, including RMSF, Queensland tick typhus, and murine typhus.^99^–^105^ Multiple retinal lesions similar to those seen in multiple white dot syndrome (MEWDS) have been reported.^106^,^107^

Rickettsial retinitis has a self-limited evolution in most patients, with resolution of white retinal lesions with or without scarring in several weeks.

#### Retinal vascular involvement

**Figure 9:**
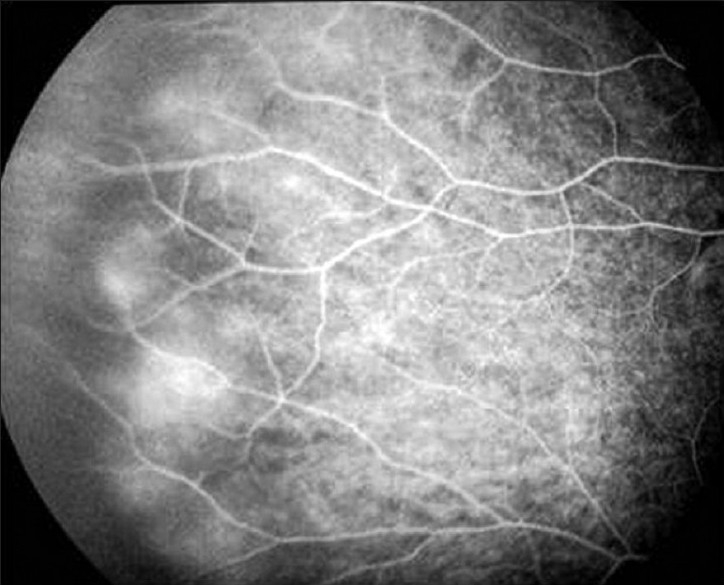
Fluorescein angiography of a patient with Mediterranean spotted fever shows retinal vascular leakage

Numerous retinal vascular lesions may occur in patients with rickettsial disease. They include focal or diffuse vascular sheathing [Figure 9], vascular leakage, intraretinal, white-centered, or subretinal hemorrhages, and retinal vascular occlusions associated with transient or permanent visual loss, including branch and central artery occlusion, and branch retinal vein occlusion or subocclusion.^97^,^98^,^108^–^111^

#### Other retinochoroidal changes

They include serous retinal detachment, cystoid macular edema, macular star, and hypofluorescent choroidal lesions on fluorescein and indocyanine green angiography.^98^,^112^

A case of endogenous endophthalmitis caused by *R. conorii* was reported recently.^113^

#### Optic nerve involvement

Optic disc involvement, with or without associated visual loss, has been described in association with rickettsial infection, including optic disc edema, optic disc staining, optic neuritis, neuretinitis, and ischemic optic neuropathy.^97^,^98^,^112^,^114^–^117^

#### Other ophthalmic manifestations

Adnexal and anterior segment manifestations of rickettsiosis include bilateral or unilateral (Portal of entry of infection) conjunctivitis, conjunctival petechia and subconjunctival hemorrhages, keratitis, nongranulomatous anterior uveitis, and iris nodule. Other reported ophthalmic findings include endophthalmitis and third and six cranial nerve palsies.

### Diagnosis

Diagnosis of rickettsial infection usually based on clinical features and epidemiologic data is confirmed by positive indirect immunofluorescent antibody test results. Positive serologic criteria usually include either initial high antibody titer or a fourfold rise of the titer in the convalescent serum. Case confirmation with serology might take two to three weeks. Other laboratory tests, such as serologic testing using western blot or detection of rickettsia in blood or tissue using polymerase chain reaction, may be useful in selected cases.

Ocular examination, revealing frequently abnormal, fairly typical findings is helplful in diagnosing a rickettsial disease, particularly in incomplete and atypical systemic presentation, while serologic testing is pending.

### Differential diagnosis

The differential diagnosis of rickettsiosis includes numerous systemic infectious and noninfectious diseases manifesting with febrile illness, such as typhoid fever, measles, rubella, enteroviral infection, meningoccemia, disseminated gonococcal infection, secondary syphilis, leptospirosis, cat scratch disease, infectious mononuleosis, arbovirus infection, Kawasaki disease, Behçet's disease and other systemic vasculitic disorders, idiopathic thrombocytopenic purpura, and drug reaction.

Other important causes of retinitis and vasculitis such as toxoplasmosis and sarcoidosis should also be considered.

### Treatment

Early treatment is critical to outcome and must be started on the basis of clinical diagnosis. Doxycycline (100 mg every 12 h for 7 to 10 days) is the drug of choice for the treatment of rickettsial diseases. Antibiotic treatment may be terminated 48 h after the patient is afebrile. Other tetracyclines (25-50 mg/Kg/day), chloramphenicol (50-75 mg/Kg/day) in four divided doses, and fluoroquninolones are also effective. Both tetracyclines and chloramphenicol have potential significant adverse effects, especially in children. Macrolides, including clarithromycin, azithromycin, and particularly josamycin can be used as an alternative therapy in children and pregnant women.^95^

Fluoroquinolones, because of their good ocular penetration, might be more effective than doxycycline in the treatment of intraocular rickettsial disease. Additional therapeutic agents may be required for ocular disease: topical antibiotic for conjunctivitis or keratitis, topical steroids and mydriatic drops for anterior uveitis, systemic steroids for severe ophthalmic involvement, such as extensive retinitis threatening the macula or optic disc, serous retinal detachment, macular edema, retinal vascular occlusion, severe vitritis, and optic neuropathy, and anticoagulant agents for retinal vascular occlusions. The role of antibiotic therapy, as well as that of oral steroids, on the course of posterior segment involvement, remains unknown. The effect of anticoagulants on the course of retinal occlusive complications is also unclear.^18^

Prevention is the mainstay for control of rickettsial diseases: personal protection against tick bites in endemic areas (repellents, protective clothing, and avoidance of dogs, detection and removal of an attached tick), improvement of sanitary conditions including the control of rat reservoirs and of flea or lice vectors.

### Evolution and prognosis

Although prognosis of systemic infection is good in most cases, rickettsioses may be severe and potentially lethal, and should be treated accordingly.

Ophthalmic manifestations of rickettsioses have a self-limited evolution in most patients, disappearing between the third and tenth week after the first examination. All inner white retinal lesions clear without causing scarring. Retinal pigment epithelium (RPE) changes develop in eyes with resolved full-thickness white retinal lesions. Retinal neovascularization developed in one patient after resolution of retinitis six months after initial presentation.^98^ Visual acuity returns to baseline in most patients. Persistent decreased vision may occur due to retinal changes secondary to macular edema or serous retinal detachment, retinal artery or vein occlusion, foveal chorioretinal scar, or optic neuropathy.
